# Dopamine use and its consequences in the intensive care unit: a cohort study utilizing the Japanese Intensive care PAtient Database

**DOI:** 10.1186/s13054-022-03960-y

**Published:** 2022-04-02

**Authors:** Reina Suzuki, Shigehiko Uchino, Yusuke Sasabuchi, Alan Kawarai Lefor, Masamitsu Sanui

**Affiliations:** 1 Department of Anesthesiology and Intensive Care, Saitama Medical Center, Jichi Medical University, 1-847, Amanuma-cho, Omiya-ward, Saitama, Zip 330-0834 Japan; 2 Data Science Center, Jichi Medical University, Shimotsuke, Japan; 3 Department of Surgery, Jichi Medical University, Shimotsuke, Japan

**Keywords:** Intensive care unit, Dopamine, Low-dose dopamine, Catecholamine, Cardiovascular, Critical care

## Abstract

**Background:**

Dopamine is used to treat patients with shock in intensive care units (ICU) throughout the world, despite recent evidence against its use. The aim of this study was to identify the latest practice of dopamine use in Japan and also to explore the consequences of dopamine use in a large Asian population.

**Methods:**

The Japanese Intensive Care PAtient Database (JIPAD), the largest intensive care database in Japan, was utilized. Inclusion criteria included: 1) age 18 years or older, 2) admitted to the ICU for reasons other than procedures, 3) ICU length of stay of 24 h or more, and 4) treatment with either dopamine or noradrenaline within 24 h of admission. The primary outcome was in-hospital mortality. Multivariable regression analysis was performed, followed by a propensity score-matched analysis.

**Results:**

Of the 132,354 case records, 14,594 records from 56 facilities were included in this analysis. Dopamine was administered to 4,653 patients and noradrenaline to 11,844. There was no statistically significant difference in facility characteristics between frequent dopamine users (*N* = 28) and infrequent users (*N* = 28). Patients receiving dopamine had more cardiovascular diagnosis codes (70% vs. 42%; *p* < 0.01), more post-elective surgery status (60% vs. 31%), and lower APACHE III scores compared to patients given noradrenaline alone (70.7 vs. 83.0; *p* < 0.01). Multivariable analysis showed an odds ratio for in-hospital mortality of 0.86 [95% CI: 0.71–1.04] in the dopamine ≤ 5 μg/kg/min group, 1.46 [95% CI: 1.18–1.82] in the 5–15 μg/kg/min group, and 3.30 [95% CI: 1.19–9.19] in the > 15 μg/kg/min group. In a 1:1 propensity score matching for dopamine use as a vasopressor (570 pairs), both in-hospital mortality and ICU mortality were significantly higher in the dopamine group compared to no dopamine group (22.5% vs. 17.4%, *p* = 0.038; 13.3% vs. 8.8%, *p* = 0.018), as well as ICU length of stay (mean 9.3 days vs. 7.4 days, *p* = 0.004).

**Conclusion:**

Dopamine is still widely used in Japan. The results of this study suggest detrimental effects of dopamine use specifically at a high dose.

*Trial registration* Retrospectively registered upon approval of the Institutional Review Board and the administration office of JIPAD.

**Supplementary Information:**

The online version contains supplementary material available at 10.1186/s13054-022-03960-y.

## Background

Since Goldberg et al. reported that administration of dopamine to patients with end-stage congestive heart failure resulted in increased cardiac output and sodium diuresis [1], dopamine has been used to treat patients with shock. It was thought that treatment with dopamine would benefit patients at risk of developing acute renal failure due to its vasodilatory effects when used at a low dose. Subsequent studies, however, refuted the beneficial effects of dopamine on patient-centered outcomes in the contexts of renal protection by 2000 [2–6] and as a vasopressor by 2012 [7–10]. It is now generally accepted that dopamine causes more harm than benefits and is not recommended for patients with at least septic shock [4, 11–13] based on the findings that dopamine is associated with an increased risk of tachyarrhythmias and possibly increased mortality compared to noradrenaline [2, 9].

Since 2012, following an epoch-making SOAP II study in 2010 and a subsequent meta-analysis, the Surviving Sepsis Campaign Guidelines continued to recommend noradrenaline as the drug of choice and recommend dopamine “only in highly selected patients (e.g., patients with a low risk of tachyarrhythmias) [14] and most recently in 2021, “in settings where norepinephrine is not available.” The guidelines 2012 and 2016 clearly state that they “recommend against using low-dose dopamine for renal protection.” In a recently published article describing online survey results from 839 members of The European Society of Intensive Care Medicine in 82 countries in 2019, the authors reported that only 2% of survey responders chose dopamine as the first choice for the treatment of patients with septic shock [15]. Despite the accumulated evidence, dopamine is still used in some countries including Japan in 2000’s [16–23]. In a study conducted to evaluate the role of polymyxin B hemoperfusion in Japanese patients with abdominal septic shock between 2007 and 2011, dopamine was given to more than 80% of patients. In a national survey conducted in China in 2012, dopamine was found to be the most preferred drug for hypovolemic shock (73.4%) and cardiogenic shock (67.1%) by the physicians. [18]. While these data suggest the continued use of dopamine, the actual patient data associated with dopamine use, e.g., type of shock, dose, in recent years are lacking.

Given the risks associated with dopamine administration discussed elsewhere, there is an urgent need to evaluate the current situation of dopamine use so that appropriate measures can be taken. In addition, it would also be important to explore the consequences of dopamine use in a large Asian population, which is never reported in the past. This retrospective analysis of the largest database of critically ill patients in Japan was conducted to identify current patterns of dopamine use and also to explore the consequences of dopamine use in this population.

## Methods

### Database

This study was approved by the Institutional Board Review at our institution as well as the administration office of the Japanese Intensive Care Case Database (JIPAD) [24]. JIPAD is the largest domestic database of critically ill patients managed by the Japanese Society of Intensive Care Medicine, which was established in 2014 and involved 89 participating facilities and 78 hospitals as of November 2020. The data were anonymized upon transfer to the database. Investigators are granted access to the data set upon approval of their request by the JIPAD administrative office. For this study, all existing case records with all data elements in the Case Report Form were requested and approved. Fifty-six facilities were included in this study, since not all the 89 participating facilities entered data in a timely manner.

### Patients

Inclusion criteria included: 1) age 18 years or older, 2) admitted to the ICU for reasons other than procedures, e.g., central line insertion, 3) ICU length of stay of equal to or more than 24 hours, and 4) use of either dopamine or noradrenaline during the first 24 hours of ICU stay. Patients fulfilling all the above criteria were included for analysis. The exclusion criterion was the presence of missing data required for multivariable analysis.

### Outcomes

Participating facilities were classified into two groups: dopamine-frequently using facilities (*N* = 28) and dopamine-infrequently using facilities (*N* = 28) based on the percentage of patients treated with dopamine with a cut-off at the median value. Stacked bar plots for facilities were made for all patients to visualize the proportion of patients treated with dopamine only, dopamine + noradrenaline, and noradrenaline only. The primary outcome was in-hospital mortality. The secondary outcomes were death at the time of ICU discharge, ICU length of stay, hospital length of stay, and duration of the first episode of mechanical ventilation.

### Variables

Data in the original dataset included: facility identification number, year, age, gender, date of hospital admission/discharge, weight, height, comorbidities (human immunodeficiency virus /acquired immune deficiency syndrome, congestive heart failure, respiratory failure, liver failure, cirrhosis, use of immunosuppressants, undergoing hemodialysis, lymphoma, acute leukemia, cancer with metastases), days before ICU admission after hospital admission, cardiac arrest leading to that ICU admission, activation of Rapid Response Team / Medical Emergency Team leading to that ICU admission, date / time of ICU admission / discharge, the reason for ICU admission (1. Transfer from the ward, 2) admission through the emergency room, 3) ICU admission following elective surgery, 4) admission following urgent surgery, and 5) other), diagnosis text, diagnosis code, Glasgow Coma Scale, the maximum serum lactate level during the first 24 hours of ICU admission, maximum / minimum laboratory values during the first 24 hours after ICU admission, the maximum value of serum bilirubin, and minimum value of platelet count, urine output during the first 24 hours after ICU admission, acute kidney injury / mechanical ventilation during the first 24 hours after ICU admission, APACHE III, SAPS II score, SOFA score, use of dopamine / dobutamine / adrenaline / noradrenaline during the first 24 hours of ICU admission, date / time of initiation and discontinuation of mechanical ventilation, ICU discharge outcome, hospital discharge outcome (alive, transferred, dead), and readmission to the ICU. Other data elements are presented in Additional file 1: Table 1. The variables collected during the first 24 hours were treated as the baseline values. A new, binary cardiovascular code was assigned when the patient had a cardiac disease code as the primary diagnosis code. A new, binary infection code was assigned when the primary disease code was one of the infection-associated codes as per the JIPAD dictionary (Last updated December 25, 2020).

**Table 1:** Study patients stratified by dopamine and noradrenaline use status

	Dopamine (*N* = 2750)	Dopamine + noradrenaline (*N* = 1903)	Noradrenaline (*N* = 9941)	*p*-value
Year = 2019 (%)	1546 (56.2)	1092 (57.4)	6100 (61.4)	< 0.001
Age (mean years, SD)	69.6 (12.4)	69.6 (12.6)	69.3 (13.6)	0.488
Gender (Male, %)	1788 (65.0)	1261 (66.3)	6507 (65.5)	0.677
Weight (mean kg, SD)	59.2 (12.7)	60.2 (13.9)	58.6 (13.9)	< 0.001
Height (mean cm, SD)	160.5 (10.3)	160.9 (10.2)	160.7 (10.1)	0.309
Comorbidities (%)				
Acquired immune deficiency syndrome	1 (0.0)	3 (0.2)	9 (0.1)	0.393
Congestive heart failure	115 (4.2)	73 (3.8)	249 (2.5)	< 0.001
Respiratory failure	29 (1.1)	17 (0.9)	236 (2.4)	< 0.001
Cirrhosis	19 (0.7)	18 (0.9)	209 (2.1)	< 0.001
Use of immunosuppressants	210 (7.6)	93 (4.9)	861 (8.7)	< 0.001
Hemodialysis	177 (6.4)	159 (8.4)	781 (7.9)	0.022
Acute leukemia or lymphoma	16 (0.6)	25 (1.3)	213 (2.1)	< 0.001
Cancer with metastases	72 (2.6)	39 (2.0)	338 (3.4)	0.002
Days before ICU after hospital admission (mean days, SD)	7.5 (17.5)	9.2 (17.7)	9.4 (27.2)	0.001
Code blue or activation of Rapid Response Team/Medical Emergency Team (%)	35 (1.3)	62 (3.3)	648 (6.5)	< 0.001
Cardiopulmonary resuscitation prior to ICU admission (%)	87 (3.2)	140 (7.4)	731 (7.4)	< 0.001
Reason for ICU admission (%)				< 0.001
Transfer from ward	153 (5.6)	257 (13.5)	2061 (20.7)	
Transfer from emergency room	310 (11.3)	296 (15.6)	2636 (26.5)	
Elective surgery	1821 (66.2)	984 (51.7)	3033 (30.5)	
Urgent surgery	376 (13.7)	287 (15.1)	1596 (16.1)	
Other	90 (3.3)	79 (4.2)	615 (6.2)	
Diagnosis code (%)				
Cardiovascular	1817 (66.1)	1421 (74.7)	4128 (41.5)	< 0.001
Valvular surgery	565	365	902	
Coronary artery bypass graft surgery	294	277	780	
Cardiac arrest	63	106	585	
Infection	93 (3.4)	207 (10.9)	2709 (27.3)	< 0.001
Post-surgery, Gastrointestinal tract perforation/rupture	34	54	508	
Bacterial pneumonia	10	24	446	
Septic shock	3	36	426	
Other	840 (30.5)	275 (14.5)	3104 (31.2)	< 0.001
Post-surgery, gastrointestinal tumor	373	43	420	
Other respiratory disease	9	10	283	
Post-surgery, oral/pharyngeal/nasal/trachea	86	9	115	
APACHE III (mean, SD)	64.9 (22.8)	79.1 (31.1)	83.0 (31.2)	< 0.001
SAPS II (mean, SD)	35.9 (14.4)	45.0 (18.7)	47.3 (18.5)	< 0.001
Mechanical ventilation during the first 24 h (%)	2198 (80)	1695 (89)	7538 (76)	< 0.001
AKI during the first 24 h (%)	63 (2.3)	128 (6.7)	786 (7.9)	< 0.001
Adrenaline during the first 24 h (%)	42 (1.5)	79 (4.2)	440 (4.4)	< 0.001
Dobutamine during the first 24 h (%)	810 (29.5)	835 (43.9)	2965 (29.8)	< 0.001

*SD* Standard Deviation, *APACHE III* Acute Physiological Assessment and Chronic Health Evaluation, *SAPS II* Simplified Acute Physiology Score. *ICU* Intensive care unit, *AKI* Acute kidney injury

### Statistical analysis

Continuous variables were presented with a mean (± standard deviation) and median (interquartile range) as appropriate. Categorical variables were reported with a number (%). For a two-group comparison, statistical significance was tested with an t-test for the continuous variables and a Chi-square test for the categorical variables. For a three-group comparison, a one-way ANOVA test was conducted for the continuous baseline variables and the Kruskal–Wallis test was conducted for the categorical variables. The baseline characteristics of patients were presented in a three-group comparison of dopamine, dopamine + noradrenaline, and noradrenaline in one table, while a two-group comparison of dopamine (dopamine + noradrenaline in dopamine group) vs noradrenaline was also conducted for an easier interpretation.

A univariable logistic regression and subsequent multivariable logistic regression analysis with a generalized estimating equation were conducted to assess the effect of dopamine use on the primary outcome utilizing “gee” package in R, accounting for differences in practice among the facilities. The primary predictor variable was a categorical variable representing 1) dopamine only, 2) dopamine and noradrenaline, and 3) noradrenaline only. The variables used for an adjustment in the multivariable analysis consisted of: age, gender (female as reference), comorbidities, days before ICU after hospital admission, cardiopulmonary resuscitation, the reason for ICU admission (others as reference), diagnosis code, APACHE III, the maximum lactate level at baseline, mechanical ventilation at baseline, AKI at baseline, dobutamine use, and adrenaline use. In the generalized estimating equation models, the dopamine variable was treated as a categorical value with four levels including:1) no use, 2) equal to or less than 5 μg/kg/min, 3) more than 5 but equal to or less than 15 μg/kg/min, and 4) greater than 15 μg/kg/min. The predictor variables in the model were selected by the study team based on their clinical importance, especially when the variables were previously reported to predict outcomes in critically ill patients including postoperative state [25], admission from the ward [26], urgent / emergent admission [27], time from hospital admission to ICU admission [28, 29], and use of mechanical ventilation [30]. The results of multivariable analysis were presented with an odds ratio with 95% confidence intervals.

To examine the specific effect of dopamine on the primary and the secondary outcomes, a propensity score-matched analysis was conducted with “Matching” package in R. To account only for the situations where dopamine was used as a vasopressor, not as an inotrope, only patients who received a vasopressor-equivalent dose of dopamine were attempted to be included. Due to unavailability of the exact dose of catecholamines for each patient due to the data entry format in JIPAD, patients in the medium and high dopamine dose categories (5–15 μg/kg/min and > 15 μg/kg/min) were included in the propensity score matching. The propensity scores for dopamine administration were calculated for study patients using a logistic regression model with the generalized estimating equation. The variables used to calculate propensity scores for treatment with dopamine are presented in the Additional file 1. The HIV / AIDS variable was removed from the multivariable model for propensity-matched cohorts as the patients with the condition were all in dopamine group. The patients with cirrhosis, acute leukemia / lymphoma, cancer with metastasis, and cardiopulmonary resuscitation prior to ICU admission were also excluded from the propensity-score matched analysis, as those conditions were considered to be associated with many other factors than dopamine treatment such as the disease type, stage, therapy, and the situation (for cardiopulmonary resuscitation). To check the balance between the two groups, the baseline characteristics were presented with standardized mean differences.

To explore the effects of dopamine on the outcomes of patients with cardiovascular disease or infection, pre-planned subgroup analyses for the propensity-matched cohorts were conducted using data from patients with a newly assigned, dichotomized primary disease code of 1) cardiovascular, 2) infection, or 3) other (neither 1 nor 2).

A *p*-value of 0.05 (bilateral) was considered statistically significant. All statistical analyses were conducted with R version 4.0.2 (R Foundation for Statistical Computing, Vienna, Austria).

## Results

### Patterns of dopamine use

**Fig. 1:**
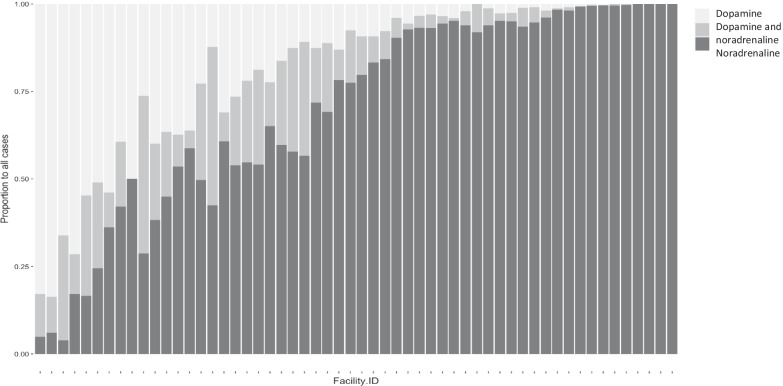
Proportion of patients treated with dopamine (light gray), dopamine and noradrenaline (gray), and noradrenaline (dark gray). X axis: Facility identification number. Y axis: Proportion of patients to all cases

Figure 1 visually represents a wide variation in percentages of patients who received dopamine (light gray), dopamine and noradrenaline (gray), and noradrenaline (dark gray) among the participating facilities (median 19.8%; IQR 4.9–48.2%, minimum 0.0%, maximum 96.2%). The median proportions of patients treated on dopamine (including those both on dopamine and noradrenaline) at each facility were 0.20 (IQR: 0.05–0.49) for all patients, 0.41 (IQR: 0.09–0.71) for cardiovascular, 0.10 (IQR: 0.04–0.26) for infection, and 0.22 (IQR: 0.08–0.48) for other diagnosis code.

The characteristics of the participating 56 facilities are presented in Additional file 1: Table 1. Public hospitals were more common among dopamine infrequent users compared to dopamine frequent users (57.1% vs 25.0%) while university hospitals were more common among dopamine frequent users (32.1% vs 53.6%). There were no statistically significant differences in the number of hospital beds or ICU beds, number of board-certified intensivists, or nurses.

### Patients’ characteristics

**Fig. 2:**
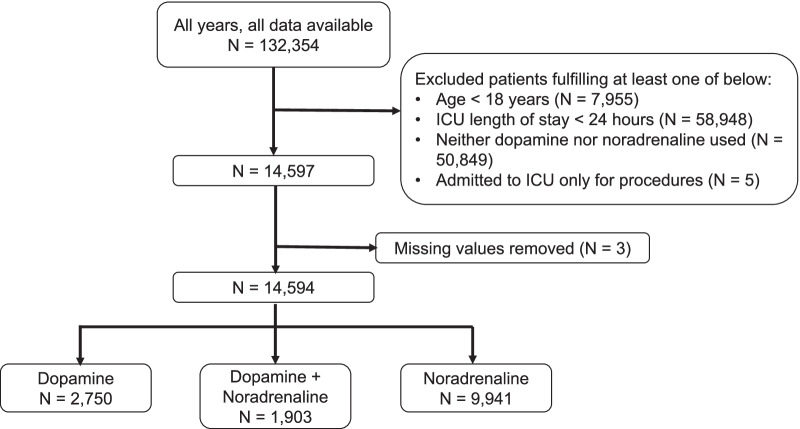
Flow diagram of the patient inclusion and exclusion process.* ICU* Intensive Care Unit

In total, 132,354 patient records dated from 2015 to 2020 were available, with 14,594 included in this analysis (Fig. 2). Dopamine was administered to 31.9% of all patients, with 13.0% receiving dopamine in combination with noradrenaline. Among patients for whom dopamine was used, 82.0% received a low dose (≤ 5 μg/kg/min), 16.9% received a medium dose (dopamine > 5 and ≤ 15 μg/kg/min), and the remainder 1.1% received a high dose (> 15 μg/kg/min). Noradrenaline was administered to 81.2% of all patients. The mean age of the patients was 69.4 years (SD 13.2) and 65.4% were male. The median ICU stay was 4.0 days (IQR: 2.5–7.5), and the median hospital stay was 29 days (IQR: 18–53). The baseline epidemiological characteristics of the patients in the three, mutually exclusive groups (dopamine only, dopamine + noradrenaline, and noradrenaline only) are summarized in Table 1 and Additional file 1: Table 2. Congestive heart failure was more common in the dopamine group compared to the noradrenaline only group (4.0% vs 2.5%, *p* < 0.01). Cardiovascular diagnosis codes were more prevalent in the dopamine group than the noradrenaline only group. Infection diagnosis codes were more prevalent in the noradrenaline only group than the dopamine group (69.6% vs 41.5%, *p* < 0.01; 27.3% vs 6.4%, *p* < 0.01, respectively). The highest APACHE III and SAPS II during the first 24 hours were higher in the noradrenaline only group than the dopamine group (83.0 vs 70.7, *p* < 0.01; 47.3 vs 39.6, *p* < 0.01, respectively). Baseline vital signs and laboratory data are presented separately in Additional file 1: Table 1.

**Table 2:** Multivariable logistic analysis with the generalized estimating equation: predictors of in-hospital mortality

Predictors	Odds ratios	CI
(Intercept)	0.02	0.01–0.03
Age	1.00	1.00–1.01
Male gender	1.04	0.95–1.04
Comorbidities		
Acquired immune deficiency syndrome	4.29	2.17–8.48
Congestive heart failure	1.77	1.38–2.28
Respiratory failure	1.44	1.06–1.95
Cirrhosis	1.60	1.22–2.09
Use of immunosuppressants	1.41	1.17–1.70
Hemodialysis	1.40	1.17–1.67
Acute leukemia or lymphoma	1.85	1.28–2.66
Cancer with metastases	1.84	1.36–2.50
Days before ICU after hospital admission	1.01	1.00–1.01
Cardiopulmonary resuscitation prior to ICU admission	1.26	0.98–1.60
Reason for ICU admission (compared to others)		
Transfer from the ward	0.79	0.66–0.93
Admission from the emergency room	0.25	0.21–0.31
Admission following elective surgery	0.67	0.56–0.81
Admission following urgent surgery	0.93	0.72–1.19
Diagnosis code (Not stated as reference)		
Cardiovascular	0.71	0.56–0.86
Infection	0.73	0.63–0.85
APACHE III score	1.03	1.03–1.03
Serum lactate level	1.05	1.03–1.06
Mechanical ventilation during the first 24 h	0.90	0.78–1.04
Acute kidney injury during the first 24 h	1.51	1.26–1.82
Dobutamine use	1.12	0.96–1.29
Adrenaline use	1.58	1.18–2.12
Dopamine use (compared to no use)		
Dopamine (≤ 5 microg/kg/min)	0.86	0.71–1.04
Dopamine (More than 5 but equal to or less than 15 microg/kg/min)	1.46	1.18–1.82
Dopamine (≥ 15 microg/kg/min)	3.30	1.19–9.19

*ICU* Intensive care unit, *APACHE III* Acute Physiological Assessment and Chronic Health Evaluation

### Associations with outcomes

A univariable logistic regression with a generalized estimating equation with death at hospital discharge as the outcome variable and dopamine as the predictor variable yielded an odds ratio (OR) 0.39 (95%CI: 0.31–0.51) for dopamine ≤ 5 μg/kg/min, OR 1.39 (95%CI: 1.11–1.73) for dopamine > 5 and ≤ 15 μg/kg/min, and OR 4.87 (95%CI: 2.85–8.34) for dopamine > 15 μg/kg/min (no use as reference). The results of multivariable logistic analysis with the generalized estimating equation are shown in Table 2. The higher doses of dopamine were still associated with a higher in-hospital mortality rate after the adjustment.

A propensity-matched analysis was conducted on the data after the inclusion process. The area under the receiver operating characteristic curve of the final model to predict the dopamine treatment was 0.73 [95%CI: 0.71–0.75]. In total, 570 pairs were made upon a 1:1 propensity score matching. The baseline characteristics of the pairs are presented in Table 3 and Additional file 1: Table 3, showing well-balanced groups with standardized mean differences < 0.1 for most of the variables***.*** The primary and secondary outcomes in these two groups are presented in Table 4. Both of the rate of death at the time of ICU and hospital discharge were significantly higher in the dopamine group. ICU length of stay and the duration of the first mechanical ventilation were significantly longer in the dopamine group by almost two days and one day, respectively. Sub-group analyses on the three specific disease categories (cardiovascular, infection, and others) did not show significant differences either in the death at the time of ICU or hospital discharge between no dopamine and dopamine groups in any of the subgroups, however, revealed a significantly longer ICU stay and the duration of the first mechanical ventilation with dopamine use in patients with a cardiovascular diagnosis. The hospital length of stay was significantly longer in the dopamine group in patients with infection. No such association was found for patients with other diagnoses.

**Table 3:** Baseline characteristics of propensity-matched groups for dopamine use

	No dopamine (*N* = 570)	Dopamine (*N* = 570)	SMD
Year = 2019 (%)	345 (60.5)	354 (62.1)	0.032
Age (mean years, SD)	70.3 (12.2)	70.6 (11.9)	0.031
Gender (Male, %)	350 (61.4)	336 (58.9)	0.050
Weight (mean kg, SD)	57.0 (13.2)	57.2 (13.9)	0.018
Height (mean cm, SD)	160.0 (10.7)	159.4 (10.8)	0.054
Comorbidities (%)			
Acquired immune deficiency syndrome	1 (0.2)	0 (0.0)	0.059
Congestive heart failure	31 (5.4)	32 (5.6)	0.008
Respiratory failure	12 (2.1)	10 (1.8)	0.026
Use of immunosuppressants	25 (4.4)	23 (4.0)	0.017
On hemodialysis	44 (7.7)	47 (8.2)	0.019
Days before ICU after admission (mean, SD)	9.6 (18.6)	10.0 (21.0)	0.019
Code blue or Rapid Response Team / Medical Emergency Team (%)	19 (3.3)	15 (2.6)	0.041
Reason for ICU admission (%)			0.081
Transfer from ward	65 (11.4)	63 (11.1)	
Transfer from emergency room	92 (16.1)	92 (16.1)	
Elective surgery	302 (53.0)	287 (50.4)	
Urgent surgery	29 (5.1)	30 (5.3)	
Other	29 (5.1)	30 (5.3)	
Diagnosis code (%)			
Cardiovascular	334 (58.6)	344 (60.4)	0.036
Valvular surgery	109	97	
Coronary artery bypass graft	67	70	
Elective surgery, aortic aneurysm	43	26	
Infection	75 (13.2)	73 (12.8)	0.010
Gastrointestinal perforation/rupture	16	20	
Bacterial pneumonia	15	8	
Septic shock	5	10	
APACHE II (mean, SD)	21.3 (8.1)	20.9 (8.0)	0.052
APACHE III (mean, SD)	81.7 (29.8)	82.5 (29.6)	0.024
SAPS II (mean, SD)	47.2 (18.4)	47.2 (18.0)	0.002
Mechanical ventilation during the first 24 h (%)	561 (98.4)	559 (98.1)	0.027
AKI during the first 24 h (%)	42 (7.4)	39 (6.8)	0.020
Adrenaline during the first 24 h (%)	45 (7.9)	43 (7.5)	0.013
Dobutamine during the first 24 h (%)	231 (40.5)	216 (37.9)	0.054
Noradrenaline during the first 24 h (%)	570 (100.0)	362 (63.5)	1.072

*SMD* Standard Mean Difference, *SD* Standard Deviation, *APACHE III* Acute Physiological Assessment and Chronic Health Evaluation, *SAPS II* Simplified Acute Physiology Score. *ICU* Intensive care unit, *AKI* Acute kidney injury

**Table 4:** Outcomes in propensity-matched analysis

	No dopamine	Dopamine	*p*-value
All patients	*N* = 570	*N* = 570	
Death at time of hospital discharge (%)	99 (17.4)	128 (22.5)	0.038
Death at time of ICU discharge (%)	50 (8.8)	76 (13.3)	0.018
ICU length of stay (mean days, SD)	7.4 (8.8)	9.3 (12.9)	0.004
Hospital length of stay (mean days, SD)	45.9 (43.7)	49.9 (50.7)	0.161
Duration of the first mechanical ventilation (mean days, SD)	4.1 (6.8)	5.0 (7.7)	0.049
Cardiovascular diagnosis	*N* = 334	*N* = 344	
Death at time of hospital discharge (%)	42 (12.6)	59 (17.2)	0.118
Death at time of ICU discharge (%)	26 (7.8)	41 (11.9)	0.094
ICU length of stay (mean days, SD)	6.8 (8.4)	10.2 (15.4)	< 0.001
Hospital length of stay (mean days, SD)	42.3 (44.4)	45.7 (39.0)	0.288
Duration of the 1st mechanical ventilation (mean days, SD)	3.1 (5.4)	4.7 (8.7)	0.007
Infection diagnosis	*N* = 75	*N* = 73	
Death at time of hospital discharge (%)	19 (25.3)	30 (41.1)	0.063
Death at time of ICU discharge (%)	9 (12.0)	16 (21.9)	0.164
ICU length of stay (mean days, SD)	9.9 (8.5)	10.6 (9.4)	0.656
Hospital length of stay (mean days, SD)	51.6 (42.7)	72.7 (81.5)	0.049
Duration of the 1st mechanical ventilation (mean days, SD)	7.1 (7.8)	7.5 (6.3)	0.708
Other diagnoses	*N* = 161	*N* = 153	
Death at time of hospital discharge (%)	38 (23.6)	39 (25.5)	0.797
Death at time of ICU discharge (%)	15 (9.3)	19 (12.4)	0.482
ICU length of stay (mean days, SD)	7.4 (9.4)	6.6 (6.2)	0.402
Hospital length of stay (mean days, SD)	50.9 (42.0)	48.4 (52.5)	0.637
Duration of the 1st mechanical ventilation (mean days, SD)	4.8 (8.1)	4.4 (5.3)	0.656

*ICU* Intensive care unit

## Discussion

These results showed that dopamine is widely used in ICUs in Japan, most commonly for patients with cardiovascular diseases. There were no significant differences in hospital characteristics between dopamine frequent-using facilities compared with infrequent-using facilities. Importantly, a dopamine dose > 5 μg/kg/min was associated with a higher probability of in-hospital mortality in a dose-dependent manner in multivariable analysis. The propensity score-matched analysis excluding those who received ≤ 5 μg/kg/min showed a significantly higher risk of death at ICU discharge or hospital discharge with dopamine use as a vasopressor. Furthermore, this analysis also showed a longer ICU stay by almost two days and a longer duration of the first mechanical ventilation by one day in the dopamine group. While a subgroup analysis showed no difference in the primary outcome associated with dopamine use in any disease categories, a significantly longer ICU stay and the duration of the first mechanical ventilation were noted with dopamine use among the patients with cardiovascular diseases.

As stated in the Surviving Sepsis Campaign Guidelines 2016, the literature on the treatment of shock after 2010—when the landmark Sepsis Occurrence in Acutely Ill Patients (SOAP) II trial was published—does not support dopamine use in treating patients with shock in general [11, 12, 31]. Despite this fact, dopamine use is still found in the literature in relatively recent years after 2010. In a nationwide survey among intensive care physicians in China in 2012, as many as 68% of the responders chose dopamine as the agent of choice for the treatment of patients with cardiogenic shock and 73% for hypovolemic shock [23]. It was found that dopamine was the most commonly used (63%) vasopressor to treat patients with traumatic shock and the second most common (28%) drug for the treatment of patients with septic shock in the Thai-shock survey 2013 [21]. Considering practice patterns in Japan, there is very limited literature on dopamine use and preliminary data were available only as incidental findings. In a retrospective study that assessed the role of postoperative polymyxin B administration in patients undergoing open abdominal surgery during 2007–2011, dopamine was used in more than 80% of patients—much higher than noradrenaline (53%) or dobutamine (10%) [32]. The other group from Japan reported in 2014 that dopamine was the most prevalently (80%) used catecholamine for patients already treated with noradrenaline in their cohort of refractory septic shock [33]. The results of the present study show that dopamine is commonly used in JIPAD participating facilities as of 2018–2019. As many as 14 out of 56 facilities used dopamine for more than 50% of patients and two of them used dopamine for > 90% of patients, suggesting a low threshold for dopamine use in some facilities. While the situation in Japan might not be applicable in other countries, these results suggest that the deleterious effects of dopamine may be underrecognized in certain situations.

In the present study, dopamine was predominantly (82%) used likely as an inotrope at a low dose (less than 5 μg/kg/min), which suggests the prevailing notion of the renal-protective effects of dopamine among physicians as of 2018–2019. While low-dose dopamine was initially expected to induce natriuresis in patients with end-stage congestive heart failure through vasodilation, the benefits of low-dose dopamine on patient outcomes were subsequently refuted in many studies. The landmark randomized controlled trial conducted by the Australian and New Zealand Intensive Care Society (ANZICS [2] revealed no reduction in the incidence of acute renal failure, the requirement for hemodialysis, or 28-day mortality with dopamine use in patients with sepsis and oliguria. Accordingly, three large systematic reviews which included this trial failed to show clinical benefits of low-dose dopamine, leading to an elimination of dopamine from clinical practice as a renal-protective agent [4–6]. The reason for the widespread use of low dose dopamine in the present study is difficult to determine, but it suggests the challenging nature of knowledge transmission or knowledge updates in clinical *practice.* The odds ratio for the hospital mortality for the low dose dopamine in the current study was lower than those for higher dose groups in the multivariable analysis (Table 2), likely reflecting the less disease severity in the patients treated with low-dose dopamine. The results were not statistically significant, however, and consistent with the study from ANZICS in 2000, which showed no mortality benefits with the low dose dopamine as described above.

While most previous studies of dopamine use focused on patients with septic shock, a recently conducted systematic review and meta-analysis with 17 trials by Hiemstra et al. included only patients with cardiac dysfunction, defined as a left ventricular ejection fraction < 45% [34]. As opposed to previous studies of patients with septic shock, the work by Hiemstra et al. did not show a significant association between dopamine use and mortality. In the patient population in the present study which consisted mostly of post-cardiac surgery patients possibly with some cardiac dysfunction, a higher mortality along with a higher dopamine dose was suggested in contrast to results in the work by Hiemstra et al. This difference could partially be explained by a difference in the dosage of dopamine used in the studies. In the cohort in the present study, only a dopamine dose more than 5 μg/kg/min was associated with higher mortality in a dose-dependent manner, while the dose range studied in Hiemstra’s work was less than in the present study (low dose: 4 μg/kg/min, moderate dose: 4–10 μg/kg/min).

Catecholamine type and doses recommended for vasoplegia in patients after cardiopulmonary bypass are not well established, though vasoplegic shock or vasoplegic syndrome is not uncommon in patients after cardiac surgery (9–44%) [33, 35, 36]. In a systematic review that assessed catecholamine use after cardiac surgery in 2007 [37], dopamine use was neither recommended nor discouraged due to a lack of evidence. A recently published consensus statement for the use of vasopressor therapy in patients undergoing cardiac surgery from Europe strongly recommends against dopamine administration for treating vasoplegic shock after cardiac surgery with an agreement of 100% [38, 39]. Although there were no randomized controlled trials which included this specific patient population in our extensive literature search, previous studies did not support dopamine use as the treatment of choice in patients after cardiopulmonary bypass [40, 41]. Considering the results in the existing literature and the results of the present study showing deleterious effects of dopamine, dopamine should not be used to treat patients after cardiac surgery or in patients with sepsis. Combined efforts at the individual level and at the facility / society level are needed to optimize dopamine use in clinical practice.

This is the largest study that assessed dopamine use and its consequences in ICUs from a single Asian country, utilizing the largest domestic critical care registry (JIPAD) in Japan. However, there are certain limitations to the present study, other than the usual limitations implicit in a retrospective study. First, patients with missing data needed for multivariable models were excluded, which might introduce selection bias. Specifically, albumin, platelet, and bilirubin variables had many missing values, leading to exclusion of these variables from the multivariable models. Second, there were no data regarding tachycardia or cardiac arrhythmias, unlike previous studies which used arrhythmias as an outcome. The available heart rate data were maximum and minimum heart rate only during the first 24 hours, which were presented as baseline data. Third, the context in which dopamine was used might not be appropriately reflected in the diagnosis code. Specifically, post-operative cardiogenic shock and post-operative vasodilatory shock were difficult to differentiate from the diagnosis codes only. Due to this problem, the specific indication for dopamine use was unclear, expecting either a renal-protective effect, vasopressor effect, or both. Fourth, the exact doses and duration of each catecholamine were not available—which might be one of the common but significant limitations of any databases requiring manual data entry. Any catecholamine requirement outside of the first 24 h window was thus not perceived in the current study. It should also be noted that the patient population in the present study seemed to be less acutely ill when compared to previous cohorts such those as in the SOAP [42] or SOAP II [7] studies, where ICU mortality was about 30% in the former and the 28-day mortality was approximately 50% in the latter—while the in-hospital mortality in the population in the present study was 18%.

## Conclusion

To the best of our knowledge, this is the largest study to describe dopamine use in patients in the intensive care unit after the SOAP II trial, utilizing a domestic database administered by the Japanese Society of Intensive Care Medicine. Dopamine was found to be used widely as of 2018–2019, most notably in patients after cardiac surgery and at a low dose. Dopamine use was associated with poor clinical outcomes in patients in this study, similar to the results of previous studies, supporting the need to change clinical practice for the use of dopamine.

## Supplementary Information

**Additional file 1. Supplementary data.** Data elements in the original data set (other than noted in the manuscript). Variables included in the generalized estimating equation model for propensity score.** Supplemental Table 1**. Baseline characteristics of hospitals stratified by frequency of dopamine use.** Supplemental Table 2**. Baseline vital signs and lab values, stratified by dopamine and noradrenaline use.** Supplemental Table 3**. The baseline vital signs and lab values of propensity-matched groups for dopamine use.

## Data Availability

The datasets generated and/or analyzed during the current study are not available since the dataset was obtained from the Japanese Intensive care PAtient Database through a formal request/approval process as above but is available from the corresponding author on reasonable request.

## Abbreviations

AKI: Acute kidney injury
APACHE: Acute physiology and chronic health evaluation
ICU: Intensive care unit
IQR: Interquartile range
OR: Odds ratio
SAPS: Simplified acute physiology score
SD: Standard deviation
