# Supplementary material for: Dopamine use and its consequences in the intensive care unit: a cohort study utilizing the Japanese Intensive care PAtient Database
Source: Crit Care. 2022 Apr 2;26:90. doi: 10.1186/s13054-022-03960-y (PMC8977005; doi:10.1186/s13054-022-03960-y)
Supplement: Supplementary file 1 — Additional file 1. Supplementary data. Data elements in the original data set (other than noted in the manuscript). Variables included in the generalized estimating equation model for propensity score. Supplemental Table 1. Baseline characteristics of hospitals stratified by frequency of dopamine use. Supplemental Table 2. Baseline vital signs and lab values, stratified by dopamine and noradrenaline use. Supplemental Table 3. The baseline vital signs and lab values of propensity-matched groups for dopamine use. [file 13054_2022_3960_MOESM1_ESM.docx]

Data elements in the original data set (other than noted in the manuscript):

Surgery text

Tracheostomy at time of ICU admission

Maximum/minimum values of vital signs during the first 24 hours after ICU admission

Maximum/minimum laboratory values during the first 24 hours after ICU admission

Maximum value of serum bilirubin and lactate during the first 24 hours after ICU admission

Minimum value of platelet during the first 24 hours after ICU admission

Urine output during the first 24 hours after ICU admission

Japan Risk of Death (JROD) predicted in-hospital mortality

Pulmonary artery catheter during the first 24 hours after ICU admission

Acute physiology and chronic health evaluation (APACHE) II

APACHE II, APACHE III, Simplified acute physiology score (SAPS) II scores-predicted mortality

Placement of arterial line

Placement of central venous catheter

Non-invasive positive pressure ventilation (NPPV)

Veno-arterial extracorporeal membrane oxygenation (VA-ECMO)

Veno-veno extracorporeal membrane oxygenation (VV-ECMO)

Intermittent renal replacement therapy (IRRT)

Continuous replacement therapy (CRRT)

Plasma exchange

Polymyxin B-immobilized fiber column hemoperfusion

Other hemofiltration

Tracheostomy

Date of tracheostomy

Intra-aortic balloon pump

Variables included in the generalized estimating equation model for propensity score

Year, age, gender, weight, height, comorbidities (each), days after hospital admission to intensive care unit (ICU) admission, code blue or Rapid response team / Medical emergency team leading to the ICU admission, cardiopulmonary resuscitation leading to the ICU admission, the reason for ICU admission, diagnosis code, Acute physiology and chronic health evaluation (APACHE) III, Simplified acute physiology score (SAPS) II, the maximum lactate level at baseline, along with the vital signs and laboratory results at baseline (maximum and minimum values of heart rate, mean arterial pressure, temperature, respiratory rate, hematocrit, white blood cell, creatinine, blood urea nitrogen, sodium, potassium, blood glucose), Glasgow Coma Score at baseline, urine output at baseline, mechanical ventilation at baseline, Acute kidney injury (AKI) at baseline, dobutamine use, adrenaline use, and quadratic terms of all the continuous variables. Bilirubin, platelet, and albumin were not added to the model due to excessive missing values.

Supplemental Table 1. Baseline characteristics of hospitals stratified by frequency of dopamine use

|  | Dopamine infrequent user  (N = 28) | Dopamine frequent user  (N = 28) | p-value |
| --- | --- | --- | --- |
| Hospital Type (%) |  |  | 0.049 |
| Private Hospital | 3 (11.1) | 6 (21.4) |  |
| Public Hospital | 16 (57.1) | 7 (25.0) |  |
| University Hospital | 9 (32.1) | 15 (53.6) |  |
| Hospital beds (mean, SD) | 751.5 (289.9) | 684.6 (245.6) | 0.356 |
| Intensive care unit beds (mean, SD) | 11.0 (5.1) | 11.7 (5.1) | 0.603 |
| Board certified intensivists (mean, SD) | 4.8 (3.1) | 4.2 (3.0) | 0.486 |
| Nurses (mean, SD) | 35.4 (13.5) | 36.6 (17.0) | 0.775 |

Supplemental Table 2. Baseline vital signs and lab values, stratified by dopamine and noradrenaline use

|  | Dopamine  (N = 2750) | Dopamine + noradrenaline  (N = 1903) | Noradrenaline  (N = 9941) | p-value |
| --- | --- | --- | --- | --- |
| Vital signs during the first 24 hours |  |  |  |  |
| HR - max | 100.1 (19.5) | 108.2 (23.4) | 111.9 (24.7) | <0.001 |
| HR - min | 68.1 (13.6) | 72.4 (15.9) | 72.14 (16.4) | <0.001 |
| MAP - max | 96.1 (15.3) | 94.2 (15.9) | 98.76 (17.9) | <0.001 |
| MAP - min | 58.7 (11.0) | 55.1 (10.5) | 55.3 (11.2) | <0.001 |
| T - max | 37.8 (0.7) | 37.8 (0.9) | 37.9 (1.0) | <0.001 |
| T - min | 36.2 (0.8) | 36.0 (0.9) | 36.2 (1.0) | <0.001 |
| R - max | 24.4 (6.0) | 24.4 (6.6) | 26.7 (7.1) | <0.001 |
| R - min | 11.3 (3.2) | 12.0 (3.4) | 12.4 (3.8) | <0.001 |
| GCS.E | 3.7 (0.8) | 3.5 (1.1) | 3.34 (1.1) | <0.001 |
| GCS.V | 4.6 (1.1) | 4.2 (1.5) | 4.06 (1.5) | <0.001 |
| GCS.M | 5.7 (1.1) | 5.3 (1.6) | 5.25 (1.6) | <0.001 |
| GCS | 14.0 (2.9) | 13.0 (4.0) | 12.7 (4.0) | <0.001 |
| Lab values during the first 24 hours |  |  |  |  |
| Hct - max | 33.0 (4.8) | 33.1 (5.1) | 33.1 (5.9) | 0.651 |
| Hct - min | 29.7 (4.8) | 29.2 (5.0) | 29.0 (5.8) | <0.001 |
| WBC - max | 11.8 (5.8) | 12.7 (6.6) | 13.5 (9.4) | <0.001 |
| WBC - min | 9.4 (5.0) | 9.7 (5.6) | 10.7 (8.2) | <0.001 |
| Cre - max | 1.5 (1.6) | 1.8 (1.7) | 1.9 (2.0) | <0.001 |
| Cre - min | 1.2 (1.3) | 1.5 (1.5) | 1.6 (1.7) | <0.001 |
| BUN - max | 22.9 (15.7) | 28.0 (18.7) | 31.7 (24.7) | <0.001 |
| Urine output during the first 24 hours | 2037.3 (1510.3) | 1697.0 (1248.8) | 1421.7 (1188.6) | <0.001 |
| Na - max | 142.5 (4.6) | 142.7 (5.7) | 141.3 (5.7) | <0.001 |
| Na - min | 137.1 (5.1) | 136.7 (5.4) | 135.9 (5.7) | <0.001 |
| K - max | 4.6 (0.6) | 4.7 (0.7) | 4.6 (0.7) | <0.001 |
| K - min | 3.7 (0.5) | 3.8 (0.6) | 3.7 (0.6) | 0.123 |
| Blood glucose - max | 197.0 (56.8) | 207.0 (72.4) | 196.8 (80.1) | <0.001 |
| Blood glucose - min | 125.6 (30.4) | 121.7 (33.7) | 116.1(36.2) | <0.001 |

Table Notes. HR: heart rate, MAP: mean arterial pressure, T: temperature, R: respiratory rate, GCS: Glasgow coma scale, GCS.E: Glasgow coma scale (eye), GCS.V: Glasgow coma scale (verbal), GCS.M: Glasgow coma scale (motor), Hct: Hematocrit, WBC: white blood cell, Cre: serum creatinine, BUN: blood urea nitrogen, Na: sodium, K: potassium. Continuous variables presented with mean(s) and SD(s).

***Supplemental Table 3. The baseline vital signs and lab values of propensity-matched groups for dopamine use***

|  | ***No dopamine***  ***(N = 570)*** | ***Dopamine***  ***(N = 570)*** | ***SMD*** |
| --- | --- | --- | --- |
| ***Vital signs during the first 24 hours*** |  |  |  |
| ***HR - max*** | ***70.4 (16.5)*** | ***71.7 (16.9)*** | ***0.080*** |
| ***HR - min*** | ***94.0 (16.9)*** | ***93.6 (15.5)*** | ***0.028*** |
| ***MAP - max*** | ***53.4 (11.0)*** | ***53.4 (11.3)*** | ***0.005*** |
| ***MAP - min*** | ***37.9 (0.9)*** | ***37.9 (0.8)*** | ***0.052*** |
| ***T - max*** | ***36.1 (0.9)*** | ***36.0 (0.9)*** | ***0.135*** |
| ***T - min*** | ***24.8 (6.8)*** | ***24.6 (6.6)*** | ***0.019*** |
| ***R - max*** | ***11.6 (3.7)*** | ***11.7 (3.6)*** | ***0.043*** |
| ***R - min*** | ***3.4 (1.1)*** | ***3.4 (1.1)*** | ***0.044*** |
| ***GCS.E*** | ***4.1 (1.6)*** | ***4.1 (1.5)*** | ***0.046*** |
| ***GCS.V*** | ***5.1 (1.7)*** | ***5.3 (1.6)*** | ***0.008*** |
| ***GCS.M*** | ***12.6 (4.3)*** | ***12.8 (4.0)*** | ***0.061*** |
| ***GCS*** | ***70.4 (16.5)*** | ***71.7 (16.9)*** | ***0.080*** |
| ***Lab values during the first 24 hours*** |  |  |  |
| ***Hct - max*** | ***32.8 (5.2)*** | ***32.9 (4.9)*** | ***0.016*** |
| ***Hct - min*** | ***28.3 (5.1)*** | ***28.9 (5.0)*** | ***0.128*** |
| ***WBC - max*** | ***12.5 (6.2)*** | ***12.3 (6.5)*** | ***0.032*** |
| ***WBC - min*** | ***9.6 (4.9)*** | ***9.2 (4.9)*** | ***0.068*** |
| ***Cre - max*** | ***1.8 (1.7)*** | ***1.8 (1.7)*** | ***0.014*** |
| ***Cre - min*** | ***1.5 (1.4)*** | ***1.4 (1.4)*** | ***0.006*** |
| ***BUN - max*** | ***26.8 (20.1)*** | ***27.4 (19.4)*** | ***0.031*** |
| ***Urine output during the first 24 hours*** | ***1614.1 (1306.7)*** | ***1598.5 (1324.8)*** | ***0.012*** |
| ***Na - max*** | ***142.8 (5.6)*** | ***143.3 (5.8)*** | ***0.075*** |
| ***Na - min*** | ***137.6 (5.5)*** | ***136.8 (5.5)*** | ***0.155*** |
| ***K - max*** | ***4.7 (0.7)*** | ***4.6 (0.7)*** | ***0.025*** |
| ***K - min*** | ***3.8 (0.6)*** | ***3.7 (0.5)*** | ***0.164*** |
| ***Blood glucose - max*** | ***208.3 (79.1)*** | ***215.0 (84.6)*** | ***0.082*** |
| ***Blood glucose - min*** | ***122.2 (40.1)*** | ***122.6 (38.9)*** | ***0.010*** |

Table Notes. SMD: standard mean difference, HR: heart rate, MAP: mean arterial pressure, T: temperature, R: respiratory rate, GCS: Glasgow coma scale, GCS.E: Glasgow coma scale (eye), GCS.V: Glasgow coma scale (verbal), GCS.M: Glasgow coma scale (motor), Hct: Hematocrit, WBC: white blood cell, Cre: serum creatinine, BUN: blood urea nitrogen, Na: sodium, K: potassium. Continuous variables presented with mean(s) and SD(s).
